# Targeting the Gut Microbiota in Chagas Disease: What Do We Know so Far?

**DOI:** 10.3389/fmicb.2020.585857

**Published:** 2020-12-10

**Authors:** Eduardo Duarte-Silva, Livia H. Morais, Gerard Clarke, Wilson Savino, Christina Peixoto

**Affiliations:** ^1^Laboratory of Ultrastructure, Aggeu Magalhães Institute (IAM), Oswaldo Cruz Foundation (FIOCRUZ-PE), Recife, Brazil; ^2^Postgraduate Program in Biosciences and Biotechnology for Health (PPGBBS), Aggeu Magalhães Institute (IAM), Recife, Brazil; ^3^Network of Immunity in Infection, Malignancy and Autoimmunity (NIIMA), Universal Scientific Education and Research Network (USERN), Recife, Brazil; ^4^Division of Biology and Biological Engineering, California Institute of Technology, Pasadena, CA, United States; ^5^Department of Psychiatry and Neurobehavioural Science, University College Cork, Cork, Ireland; ^6^APC Microbiome Ireland, University College Cork, Cork, Ireland; ^7^National Institute of Science and Technology on Neuroimmunomodulation (INCT-NIM), Oswaldo Cruz Institute, Oswaldo Cruz Foundation, Rio de Janeiro, Brazil; ^8^Laboratory on Thymus Research, Oswaldo Cruz Institute, Oswaldo Cruz Foundation, Rio de Janeiro, Brazil

**Keywords:** Chagas disease, *Trypanosoma cruzi*, gut microbiota, prebiotics, probiotics

## Abstract

Chagas disease (CD) is a tropical and still neglected disease caused by *Trypanosoma cruzi* that affects >8 million of people worldwide. Although limited, emerging data suggest that gut microbiota dysfunction may be a new mechanism underlying CD pathogenesis. *T. cruzi* infection leads to changes in the gut microbiota composition of vector insects, mice, and humans. Alterations in insect and mice microbiota due to *T. cruzi* have been associated with a decreased immune response against the parasite, influencing the establishment and progression of infection. Further, changes in the gut microbiota are linked with inflammatory and neuropsychiatric disorders, comorbid conditions in CD. Therefore, this review article critically analyses the current data on CD and the gut microbiota of insects, mice, and humans and discusses its importance for CD pathogenesis. An enhanced understanding of host microbiota will be critical for the development of alternative therapeutic approaches to target CD, such as gut microbiota-directed interventions.

## Introduction

Chagas disease (CD), also known as American trypanosomiasis, is a malady that affects >8 million people worldwide (Lidani et al., 2019) resulting in high socioeconomic burden to our society (Lee et al., 2013). Although it was discovered more than 100 years ago by Carlos Chagas and comes with a high health burden (Chagas, 1909; Lee et al., 2013), it continues to be a neglected disease (Schofield et al., 2006; Clayton, 2010). The flagellate protozoan *Trypanosoma cruzi* (*T. cruzi*) is the causative agent of CD, and it is primarily transmitted to humans and animals via insect vectors known as triatomines. CD is more prevalent and endemic in Latin American countries. However, more recently, other non-endemic areas, such as United States, Canada, Europe, Australia, and Japan are starting to be affected by CD due to increased immigration world-wide (Gascon et al., 2010; Jackson et al., 2014). It is thought that CD burden could be attenuated with disease control approaches, including vector control and treatment of the infection at an early stage (Schofield et al., 2006; Gascon et al., 2010).

Infection symptoms include inflammation in the gastrointestinal tract (GIT) and heart dysfunctions and may also include neurological and behavioral disturbances (Prata, 2001; Marchi and Gurgel, 2011; Ozaki et al., 2011; Vilar-Pereira et al., 2015; Pérez-Molina and Molina, 2018). Interestingly, recent evidence now suggests an involvement of other biological factors that may contribute to mechanisms underpinning CD pathophysiology. For instance, the role of the gut microbiota in CD has been reported by preclinical and clinical studies (Duarte et al., 2004, 2005; McCall et al., 2018; Robello et al., 2019; De Souza-Basqueira et al., 2020). Gut microbiota alterations have been found in triatomine insects, mice, and human hosts (Duarte et al., 2004, 2005; Garcia et al., 2007; Díaz et al., 2016; McCall et al., 2018; Robello et al., 2019). For example, alterations in the gut microbiota of triatomine insects, including changes in the Enterobacteriaceae and Nocardiaceae family as well as low CFU counts, may increase the susceptibility to infection by impairing immune response against *T. cruzi* (Castro et al., 2012; Vieira et al., 2016). It is possible that gut microbiota alterations induced by *T. cruzi* may aggravate the host’s pathology due to modulation of the immune system (Díaz et al., 2016). However, the impact of the gut microbiota on CD’s pathophysiology remains to be fully understood. In this review, we critically analyze the current data associating CD and the gut microbiota and the importance of this interaction for CD pathogenesis. An enhanced understanding of this relationship will be critical for the development of alternative therapeutic approaches for CD treatment.

## Chagas Disease Transmission and Infection Cycle

**FIGURE 1:**
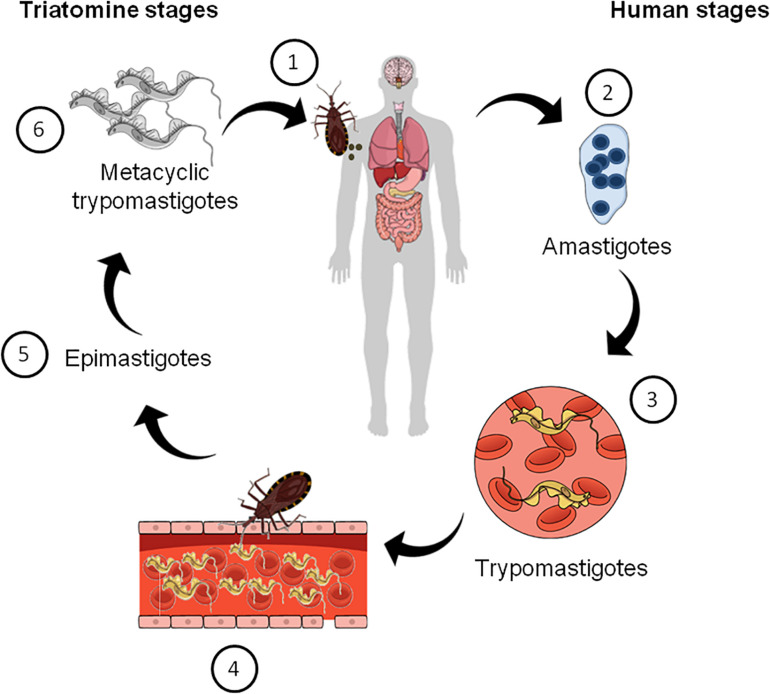
Schematic summarizing the life cycle of *Trypanosoma cruzi*. (1) Triatomine bugs take a blood meal and passes *metacyclic trypomastigotes* in their feces, which then enter the host via bite wounds or mucosal membranes. Metacyclic trypomastigotes now invade host cells (not shown) and become *amastigotes*, which multiple inside the infected cells (2). Amastigotes then transform into *trypomastigotes*, which then cause cell lysis and their release into the bloodstream (3). Triatomine bugs become infected when they feed on an infected mammalian host (4). Inside the insect vector, trypomastigotes now transform into *epimastigotes* in the insect gut (5). Finally, epimastigotes transform into infective *metacyclic trypomastigotes* (6), which are released in the feces and the cycle re-starts.

The mode of CD transmission varies according to geography (Lidani et al., 2019). In Latin America, it is mainly transmitted via insect vectors known as triatomines, which are infected with the parasite after a blood meal from infected humans or other animals, according to the parasite’s life cycle (Figure 1). In non-endemic places, blood transfusion, organ donation, congenital transmission during pregnancy, or via oral route with contaminated food and water are the main modes of CD transmission (Lidani et al., 2019). After infection with *T. cruzi* the disease can follow two distinct phases. In the acute phase, an increased parasitemia is observed. Although at this stage the disease is usually asymptomatic, there may be a few symptoms, such as fever, inflammation, tachycardia, fatigue, which can spontaneously disappear in most patients (Prata, 2001; Clayton, 2010; Pérez-Molina and Molina, 2018; Lidani et al., 2019). The chronic phase of the disease affects one third of the patients and begins with a latency period known as the indeterminate form of CD. This can persist for more than 30 years or throughout life unnoticed. After that phase, some patients can develop a symptomatic phase in which a decline in parasitemia and neurological, cardiac and digestive manifestations are observed as well as neuropsychiatric comorbidities and behavioral changes (Prata, 2001; Hueb and Loureiro, 2005; Clayton, 2010; Marchi and Gurgel, 2011; Ozaki et al., 2011).

## The Gut Microbiota

The gut microbiota comprises a community of bacteria, archaea, fungi, and viruses that has co-evolved with their hosts over thousands of years to form intricate commensal relationships (Rinninella et al., 2019). The bacterial cell number present in the human gut has been estimated to be around 3.8 × 10^13^, which is similar to our cell number in the body (Sender et al., 2016). Although the number of bacterial species is high, most of them belong to Firmicutes, Bacteroidetes, and Actinobacteria phyla (Falony et al., 2016). The gut microbiome interacts with host essential physiological processes such as modulation of immune system, metabolism, and neurotransmission, which ultimately coordinate host homeostasis (Keely, 2017). Regarding immunity, it is known that the gut microbiota modulates the development of CD8^+^ T cells, lymphocytes with key roles in the control of *T. cruzi* (Martin and Tarleton, 2004; Acosta Rodríguez et al., 2019). Gut microbiota changes with antibiotic treatment were associated with altered cytokine response and T cell receptor (TCR) signaling in CD8^+^ T cells (Gonzalez-Perez and Lamousé-Smith, 2017). Furthermore, certain strains of gut bacteria are able to induce CD8^+^ T cells in the intestine, which is associated with enhanced immunity against *Listeria monocytogenes* and enhanced anti-tumor activity (Tanoue et al., 2019). The contribution of the gut microbiome to human health and disease continues to be unraveled. However, in the recent years, large-scale studies using emerging technologies in microbiome research, including 16S ribosomal RNA (rRNA) sequencing for taxonomic characterization and whole genome shotgun (WGS) metagenomic sequencing for genomic and metabolic functional analysis are accelerating the discovery of new links between the gut microbiome, human health, and disease (Kho and Lal, 2018).

The gut microbiota signals to their host using metabolic products, neurotransmitters, cytokines, and anti-microbial substances (Fischbach and Segre, 2016). On the microbiota side, one of the most studied mediators of this communication are bioactive molecules named short-chain fatty acids (SCFAs), which consist of bacterial-derived dietary fermentation products. The most common SCFA are acetate, propionate, and butyrate. SCFAs can modulate host cell functions by controlling gene transcription through epigenetic pathways, through the activation of “metabolite-sensing” G-protein coupled receptors (GPCRs) or indirectly via interactions with host’s energetic metabolism and immune system (Wilson, 2005; Smith et al., 2013). Importantly, acetate, the most abundant SCFAs present in the blood circulation, influences cardiac function such as blood pressure and heart rate in mice via at least two modes: renin release in the juxtaglomerular apparatus and changes in vascular tone in the periphery (Onyszkiewicz et al., 2020). Furthermore, butyrate can inhibit histone-deacetylases (HDACs), affecting the gene expression of CD8^+^ cytotoxic T cells (CTLs) and causes an increase in the expression of interferon-γ (IFN-γ) and granzyme B in this cell population (Luu et al., 2018).

Another key mediator factor between the gut microbiota and their hosts is the immune system. The gut microbiota is known to direct the immune system maturation, development, and response. Specific gut microorganisms named segmented filamentous bacterium, non-culturable Clostridia-related species, guide the development of hosts IL-17 (Th17) and IFNγ (Th1) T cells in the gut mucosal immune system of rodents (Gaboriau-Routhiau et al., 2009; Hooper et al., 2012). Further, colonization of germ-free (GF) mice (mice born and raised without microbiome) with commensal microbiota induces IgA in intestinal dendritic cells, further affecting hosts defense against non-pathogenic and pathogenic microorganisms. Microbial signaling to host immune system is also facilitated by microbial metabolites and by bacterial products such as SCFAs and polysaccharide (PSA), among others (Kim et al., 2013; Macia et al., 2015). From the host side, signals are sent via hormones, cytokines, anti-microbial products, which will further change gut microbial communities’ structure and function (Fischbach and Segre, 2016). Thus, it is not surprising that such intricate relationship between the gut microbiota and their hosts are implicated in many diseases, ranging from inflammatory and cardiovascular diseases, metabolic, psychiatric, and neurological conditions. The bidirectional communication between the microbiota and other physiological systems of the body, such as the nervous, endocrine, and immune system collectively form the *microbiota-gut-brain axis* (Cryan et al., 2019). This constant crosstalk allows the host to maintain homeostasis of essential physiological processes, such as neurotransmission, appetite, neuroprotection, neurogenesis, which ultimately coordinate behavior. Data now available demonstrate that dysfuncion in the gut microbiota homeostasis play a role in many chronic inflammatory, neuroinflammatory, and neuropsychiatric disorders (Burokas et al., 2015; Sherwin et al., 2016; Sandhu et al., 2017). However, the role of the gut-microbiota-brain axis in CD remains largely unknown. Therefore, the exploring its role could provide new insights into CD pathophysiology and treatment.

## The Gut Microbiota in Chagas Disease

Microorganisms that inhabit the gut of vector insects have a pivotal role in the modulation of vector competence, which is “the ability to acquire, maintain, and transmit pathogens” (Lane, 1994). These microbes can interfere with vector competence either directly or indirectly (Dillon and Dillon, 2004; Azambuja et al., 2005; Garcia et al., 2007, 2010; Cirimotich et al., 2011; Weiss and Aksoy, 2011). In the first case, they interact with parasites and compete for resources present in the triatomine gut. In the second case, the triatomine gut microbiota can trigger anti-parasitic mechanisms and immune responses against the parasite, modulating parasite transmission (Dillon and Dillon, 2004; Azambuja et al., 2005; Garcia et al., 2007, 2010; Cirimotich et al., 2011; Weiss and Aksoy, 2011). A study that characterized triatomine bacterial communities demonstrated that *T. cruzi* infection changes the gut microbiota of these insects and it depends on “the intrinsic qualities” of the parasite itself, the insect vector and the gut microbiota of the host (Díaz et al., 2016). In the laboratory setting, the triatomine gut microbiota is characterized by low diversity of microbial population and dominance of one or few genera and specificity of bacteria to some triatomine hosts, which means that some genera of bacteria are found in specific hosts but not all host species (Díaz et al., 2016).

## Gut Microbiota Alterations in Clinical and Experimental CD: A New Player in CD Pathogenesis?

### Vector–Parasite–Microbiota Interaction in Mice and Humans

Over the years, studies focusing primarily on the parasite-vector and parasite-host cell interaction have been performed, and much insight regarding *T. cruzi* infection establishment and disease progression was gained following this approach (De Souza et al., 2010; De Oliveira et al., 2018). Nonetheless, given the crucial role the microbiota has to the host itself, the studies herein discussed focused on a tripartite interaction known as the “vector–parasite–microbiota interaction,” which is an approach that considers the gut microbiota of the host as relevant to disease establishment and progression (Díaz et al., 2016). The idea that gut microbiota was somehow linked to CD was previously hypothesized in a series of studies using GF mice. An early study comparing the impact of acute Y strain of *T. cruzi* infection on GF mice and conventional mice demonstrated that the absence of gut microbiota in the GF mice leads to a much more severe course of infection (Silva et al., 1987). A possible reason for these outcomes is that, as showed in another study, acutely infected mice also had an impaired cellular and humoral-mediated immune response against the parasite, as observed by lowered levels of IFN-γ, TNF-α, nitric oxide (NO), and antibodies specifically generated against *T. cruzi* antigens (anti-*T.cruzi* IgG1 and IgG2a) (Duarte et al., 2004). These findings are in keeping with the fact that the microbiome educates and shapes the immune system (Lee and Mazmanian, 2010; Hooper et al., 2012).

Consequently, it is expected that GF mice have an immature immune system and therefore display an altered immune response to an immune challenge. It should be noted that these studies do not offer a causal association between the gut microbiota and CD, but rather demonstrate the importance of the gut microbiota on priming the immune response in the context of parasitic diseases. Whether specific members of the microbial community contribute to the modulation of CD remains to be determined.

In a preliminary attempt to answer this question, a sequence of studies investigated the role of specific gut bacteria on mediating immunomodulatory effects in the host following acute exposure to *T. cruzi* (Silva et al., 1987; Duarte et al., 2004). In a study, GF Swiss mice received a single intragastric and individual injection of *Escherichia coli*, *Enterococcus faecalis*, *Bacteroides vulgatus*, or *Peptostreptococcus* sp. 10 days prior to infection with 5 × 10^3^ *T. cruzi trypomastigotes* (Y strain) (Please refer glossary). The authors found that *gnotobiotic mice* had increased survival rates when compared to control GF mice (Duarte et al., 2005), which tend to die earlier after infection (Silva et al., 1987). Conversely, a separate study demonstrated that monocolonization with *Bacteroides fragilis*, *Clostridium* sp., and even *Peptostreptococcus* sp. were associated with earlier mortality in acute experimental CD (Barros et al., 1992). The results for *Bacteroides fragilis* are undoubtedly surprising, since polysaccharide A (PSA) derived from this bacterium has modulatory effects on the immune system, driving its maturation and balance of Th1/Th2 responses in mice (Mazmanian et al., 2005), which would be ideal for *T. cruzi* infection resolution. The reasons for these apparently contradicting data are hitherto undefined, buy may be due to a more Th2-associated response, which consequently promotes parasite’s persistence in the host.

Interestingly, increased levels of NO were only observed in *E. coli* and *Peptostreptococcus* sp.-associated mice, while the lowest and highest production of IgG1 and IgG2a levels, respectively, were only shown in *Peptostreptococcus* sp.- associated mice. Furthermore, only mice that received *E. faecalis* had a rise in IL-10 in cultured splenocytes. These findings suggest that some of the observed immunomodulatory effects depends on certain bacterial species and cannot be generalized (Duarte et al., 2005). In fact, immunomodulation is a well recognized feature of commensal gut bacteria, contributing not only to mucosal immunity, but also to immune tolerance (Donaldson et al., 2015; Libertucci and Young, 2019). Collectively, these data demonstrate that colonization of the gut with specific bacteria strains normalizes the immune function, allowing the host to respond to *T. cruzi*.

The studies so far reported only showed that the gut microbiota has a role in immune homeostasis, which is important for the resolution of acute infection with parasites in general. However, a recent study using male C3H/HeJ mice infected with a luciferase-expressing *T. cruzi* strain (CL Brener) demonstrated a more direct association between *T. cruzi* infection and gut microbiota alterations. In this study, the authors sampled fecal pellets twice a week during the acute phase and every 2–3 weeks during the chronic phase. Using 16s rRNA sequencing it became evident that *T. cruzi* infection was associated with changes in *Bacteroidales* and *Clostridiale*s order, which belong to *Bacteroidetes* and *Firmicutes* phyla. Further, host fecal metabolic status was assessed using ultra-high performance liquid chromatography tandem mass spectrometry (UHPLC-MS/MS) and revealed alterations in fatty acids and bile acids metabolism in mice infected with the parasite (McCall et al., 2018).

More pronounced differences between groups were found at 21-days post-infection. Linoleic and linolenic acids are fatty acids metabolized by gut bacteria, including by members of the family *Ruminococcaceae* and *Lachnospiraceae* (phylum *Firmicutes*) that are present in the mice cecum and feces (Gu et al., 2013), into conjugated linoleic and linolenic acid and other derivative molecules, such as vaccenic acid (Zhang and Davies, 2016). These metabolites have been associated with anti-inflammatory response in other disease models such as animal models of colitis (Bassaganya-Riera et al., 2004; Miyamoto et al., 2015) and colorectal cancer (Evans et al., 2010). For instance, in colitis animal models, linoleic acids can act locally by downregulating the TNF-α receptor (Miyamoto et al., 2015) and TNF-α expression (Bassaganya-Riera et al., 2004) and upregulating production of anti-inflammatory cytokine TGF-β in the colon (Bassaganya-Riera et al., 2004). Moreover, they decrease the infiltration of immune cells in the colon of mice with colorectal tumors, while increasing the number of regulatory T cells (Tregs) in the mesenteric lymph node (Evans et al., 2010).

Thus, a plausible possibility is that changes in the gut microbiome composition and consequently fecal metabolite alteration may favor *T. cruzi* survival through inducing an anti-inflammatory response in the host (McCall et al., 2018). However, whether these outcomes are relevant to humans with CD is still a matter of debate. Moreover, primary bile acids are produced by the host and further modified by the gut bacteria, including members of Clostridiales order or from the genus *Bifidobacterium* or *Lactobacillus*, to generate secondary bile acids (2BAs) such as deoxycholic acid. Changes in the metabolism of bile acids are associated with inflammation in the GIT (Gérard, 2013), but its role in CD remains poorly understood. Taken together, experimental CD leads to changes in bacteria that modulate fatty acids and bile acid metabolism in mice and these fecal microbiome and metabolome changes may be relevant for the persistence of *T. cruzi* in the host.

A recent study was conducted with twenty Bolivian children who were diagnosed with CD and treated with benznidazole, a CD first line treatment drug (Robello et al., 2019). Fecal samples were obtained before and after treatment and 16s rRNA sequencing was used to analyze the microbiota and uninfected subjects were used as controls. It was observed that the parasite induced changes not only in the gut microbiota of these individuals, but also in the skin microbiota. *T. cruzi* infection was associated with high amounts of fecal *Prevotella* (phylum Bacteroidetes), *Ruminococcaceae* and *Succinivibrio* (phylum Proteobacteria). In humans, *Prevotella* was associated with increased plasma levels of trimethylamine-*N*-oxide (TMAO), a molecule implicated in cardiovascular disease (Koeth et al., 2013). Overall, infected children had increased Firmicutes and lowered Bacteroides, despite variations in age and diet (Robello et al., 2019).

Another study employing next generation sequencing (NGS) to investigate the gut microbiota composition of one hundred and fourteen Brazilian individuals with different forms of chronic CD also confirmed that *T. cruzi* triggers human gut microbiota changes (De Souza-Basqueira et al., 2020). In this study, thirty patients had the cardiac form, eleven had the digestive form, thirty-two had the indeterminate form and thirty-one were healthy individuals. The authors found lowered levels of Verrucomicrobia phylum as well as decrease in Veillonellaceae family (phylum Firmicutes) and *Dialister* genera (phylum Firmicutes) in patients with cardiac CD. Indeterminate CD patients had lowered Bacteroidaceae family (phylum Bacteroidetes), specifically Bacteroides genera when compared to controls. Patients with digestive form of CD and megacolon had reduction in Lachnospiraceae family but increase in Porphyromonadaceae family (phylum Bacteroidetes) (De Souza-Basqueira et al., 2020).

Overall, all the main gut bacteria phyla play a prominent role in colonization resistance, but some members may also be involved in inflammation. For example, *Lactobacillus* spp. from the Firmicutes phylum are able to inhibit *C. difficile* colonization and reduce inflammation, while segmented filamentous bacteria (SFB), also from the Firmicutes phylum, induce the secretion of antimicrobial peptides (AMPs), pro-inflammatory cytokines and IgA, as well as trigger the development of CD4^+^ T helper 17 (T_*H*_17) cells (Buffie and Pamer, 2013). Besides, studies now suggest that changes in the gut microbiota community may be associated with increased infection susceptibility (Libertucci and Young, 2019). However, since our understanding of the microbiota in CD is still in its infancy, studies are yet required to clarify the role of certain microorganisms in determining higher susceptibility to infection or even infection clearance, since many of them may present trypanolytic activity and therefore are vital for CD modulation.

Taken together, the aforementioned data show that *T. cruzi* infection is followed by changes in the gut microbiome and metabolome, which might be important for the parasite persistence in the host. Besides, the importance of other microbiota (oral and skin) to the pathogenesis of CD is also highlighted and deserves to be more defined in future studies. Nonetheless, these studies are preliminary and did not explore the *consequences* of these gut microbiota changes in the human population.

### Vector–Parasite–Microbiota Interaction in Insects

Studies performed with triatomine vectors are also important to our current understanding of the role of the gut microbiota in CD. For example, a study was conducted to determine the microbiota changes that results from *T. cruzi* infection in *Rhodnius prolixus*, a vector of *T. cruzi* (Castro et al., 2012). In this study, *R. prolixus* were fed rabbit blood with or without the Dm28c clone of *T.cruzi*, which can complete its developmental cycle in the insect gut (Vieira et al., 2016). After insects were fed with blood, analyses of the gut microbiota using colony forming unit (CFU) assay were performed from day 5 to 29, as well as antibacterial and phenoloxidase (PO) assays and NO measurement. Insects that were infected with *T. cruzi* presented low gut microbiota population as revealed by less CFU numbers in the agar plates (Vieira et al., 2016). Interestingly, a similar study using the same *T. cruzi strain* showed that after infection there were lower CFU counts and reduced numbers of *Serratia marcescens* (Enterobacteriaceae family), a bacterium with trypanolytic activity (Da Mota et al., 2019). Furthermore, reduced numbers of *Rhodococcus rhodnii* were also reported (Vieira et al., 2016). This bacterium belongs to Nocardiaceae family and helps in the processing of B vitamins in the triatomine’s gut (Rodríguez et al., 2011; Pachebat et al., 2013).

Accordingly, another study showed that infection of six different species of *T. cruzi* vectors (*P. megistus*, *R. prolixus*, *T. brasiliensis*, *T. infestans*, *T. juazeirensis*, and *T. sherlocki*) with *T. cruzi epimastigotes* strain 0354 resulted in changes in gut microbiota community depending on the studied hosts, in a “species-specific manner,” as well as in increased diversity in gut microbiota as demonstrated by 16s rRNA sequencing (Díaz et al., 2016). Furthermore, the gut antimicrobial activity was increased in *T.cruzi*-infected insects, as measured by inhibition zone and turbidometric assay (Castro et al., 2012). However, the antimicrobial activity did not result in the parasite’s elimination and it is rather explained by reduction in the microbial population, leading to low microbiota population (Castro et al., 2012). Moreover, the activity of PO, an important enzyme of the innate immune system of insects, and NO, an immune system mediator, were also investigated (Castro et al., 2012). The authors found that, PO activity levels were elevated and NO was decreased in infected insects. However, the increase in PO did not correlate with reduction of parasite density in the gut (Castro et al., 2012). Although PO activity levels were not enough for killing the parasite, it may be linked with changes in gut microbiota composition observed in the vector insects. These studies highlights that (i) *T.cruzi* interacts with triatomine vector microbiome and causes changes in the microbiome; (ii) the microbiome seems to be important for control of *T. cruzi*, since antibiotic treated insects had higher parasite density in the gut (Castro et al., 2012); (iii) vectorial competence may be associated with microbiota profile of vector insects; (iv) the microbiota changes observed after *T.cruzi* infection alter vector immunity.

In sum, insects studies show that *T. cruzi* infection is associated with changes in the gut microbiota, especially reduction in bacterium with trypanolytic activity and increased diversity of gut microbiota. *T. cruzi* infection also increased the secretion of AMPs, leading to a reduction in the gut microbiota community, rather than eliminating the parasite.

## Microbiota, CD, and Its Comorbidities

As already discussed, heart inflammation is a key feature of CD. Interestingly, data are now suggesting that microbial-derived molecules and metabolites, such as microbe-associated molecular patterns (MAMPS), SCFAs, 2BAs, and TMAO may underpin cardiovascular disease (CVD) (Howitt and Garrett, 2012; Zhang and Davies, 2016; Brown and Hazen, 2018). Interestingly, some gut microbes can produce trimethylamine (TMA), which results from the metabolism of fat-rich nutrients by enzymes known as microbial TMA lyases. After being produced, TMA can then enter the portal circulation and the liver, where it is further processed to generate TMAO, which triggers heart and kidney dysfunction, such as atherosclerosis, heart failure, renal fibrosis (Brown and Hazen, 2018). Furthermore, studies now acknowledge that CD patients frequently suffer from other comorbid conditions, such as major depressive disorder (MDD) and anxiety (Hueb and Loureiro, 2005; Marchi and Gurgel, 2011; Ozaki et al., 2011; Jackson et al., 2012; Suman et al., 2017). Unsurprisingly, the gut microbiome has been associated with the development of these neuropsychiatric disorders (Mayer et al., 2014; Sharon et al., 2016; Dinan and Cryan, 2017; Cryan et al., 2019). It should be noted that CD also affects the enteric nervous system (ENS) (Meneghelli, 1985; Iantorno et al., 2007). Unsurprisingly, the gut microbiome has been recognized to take part in the modulation of ENS development and function (Obata and Pachnis, 2016; Obata et al., 2020) and the ENS is able to modulate the gut microbiome (Rolig et al., 2017). Based on the data mentioned above, one could speculate on whether the gut microbial community would be an additional underpinning mechanism behind mood, cardiovascular, and ENS changes in CD. Nevertheless, this needs to be further investigated and corroborated.

## Emerging View of CD on the Light of the Microbiota: How Does the Gut Microbiota Change Our Current View of CD?

As discussed in the introduction section, CD was previously considered a disease resulting only from the parasite-host interaction, but currently this view is changing due to the inclusion of the host-microbiome as new partner in this intricate relationship. As a result, studies are now focusing on the “*vector–parasite–microbiota* interaction” (Díaz et al., 2016) and new insights on the pathogenesis of CD are consequently emerging. Unfortunately, the data collected so far does not precisely draw a new picture of CD, but they indeed point toward a new direction researchers should look at when designing new experiments. This fact is relevant because these new studies will probably lead to the discovery of new bacteria with essential activities for modulation of *T. cruzi* infection and infection resolution. At a later stage, these newly discovered bacteria may be manipulated either pharmacologically or genetically (Taracena et al., 2015) to benefit the host directly or indirectly. Identifying bacterial communities present in the human microbiome that can act against *T. cruzi* is a task that remains unresolved and therefore, should be urgently addressed.

Throughout this review, we could observe that *T. cruzi* infection results in alterations in the gut microbiota of triatomine vectors, and this allows the parasite to progress and establish infection. Later, when these vectors are in contact with humans or animals and transmit the parasite to these new hosts, *T. cruzi* again induces changes in their gut microbiota. Here, we postulate that the same process that happens in insects occurs in humans or mice: the parasite induces changes in bacteria population. These changes are responsible for alterations in the fecal metabolome, impaired immune response, and poor resolution of disease, leading to its persistence in the host. If that is the case, then the parasite uses the same *modus operandi* for insects and human hosts. Nevertheless, this hypothesis still needs to be confirmed by future studies.

## New Perspectives on the Treatment of CD: Focus on Prebiotics and Probiotics

Benznidazole and nifurtimox are the only available drugs used to treat CD over the last 40 years. They are especially effective in curing the disease when employed in the acute phase. However, their effectiveness declines when they are used during the chronic phase (Clayton, 2010). One of the main problems regarding CD treatment is the *timing* and *side effects* of current therapies (Clayton, 2010). For instance, a period of 60 days is required for benznidazole to cure the disease at the early stage and this drug usually has severe side effects (Clayton, 2010). This makes adhesion to treatment and prescription of these drugs a big issue that needs to be urgently tackled. Interestingly, benznidazole may improve CD in association with gut microbiota modulation, as infected children treated with this drug had decreased *Prevotella* and *Coprococcus* (phylum Firmicutes), increase in the amount of *Dialister* (phylum Firmicutes) and *Enterobacteriaceae* (phylum Proteobacteria) (Robello et al., 2019).

The fact that gut microbiota is somehow related to CD etiopathogenesis undoubtedly opens up the possibility of development and the use of new treatments for CD. In this regard, *prebiotics* – non-digestible fibers metabolized by the gut microbiota – and *probiotics* – living organisms that may promote health benefits – deserve special focus since they are already being tested preclinically for other diseases, such as irritable bowel syndrome (IBS) (Başturk et al., 2016; Chen et al., 2017; Ford et al., 2018; Trifan et al., 2019), although the results are still preliminary. Regarding CD and the use of these treatments, there is hitherto only one report using probiotic and none using prebiotics. Therefore, whether they may be a valid and efficacious treatment for CD will only be confirmed in the future. However, probiotics as therapeutic agents against parasitic diseases have long been proposed (Travers et al., 2011).

Gut microbiota-directed interventions hold promise for CD treatment since some genres of bacteria can modulate immunity, which is known to be impaired in CD patients. By its immunomodulatory effects the host may be able to mount a more potent immune response against *T. cruzi*, but whether this is effective in preventing or eliminating infection is still largely unknown and begs more research in the future. In fact, only one study showed positive effects of probiotics in experimental CD. *Lactobacillus casei* was administered either orally or intraperitoneally to Swiss female mice 7 days prior to infection with *T. cruzi* (Ninoa strain) and the authors observed decreased parasitemia in the groups that received probiotics (Garfias et al., 2008). This study raises many important issues that deserve to be addressed: (a) the authors used a preventive or prophylactic approach, in which probiotics were given before infection; however, to determine what effects probiotics have in infected subjects, studies also need to focus on the therapeutic approach, in which probiotics are given after infection; (b) probiotics administration led to reduced number of blood parasites, not to the complete elimination of parasites from the host and therefore they may not be suitable to be used as monotherapy in the treatment of CD. Probiotics may, however, be useful as co-therapies with standard drugs, but this still needs to be tested. Therefore, more studies with pre- and probiotics are required, especially because, once their effectiveness is proven experimentally and in the clinical setting, patients may benefit from the fact that these are low-cost and non-invasive therapies, with fewer side effects than the current drugs used to treat CD. In sum, an in-depth understanding of the gut microbiota in CD will allow us to develop alternative therapeutic approaches to target CD, such as gut microbiota-directed interventions.

## Concluding Remarks and Future Perspectives

**FIGURE 2:**
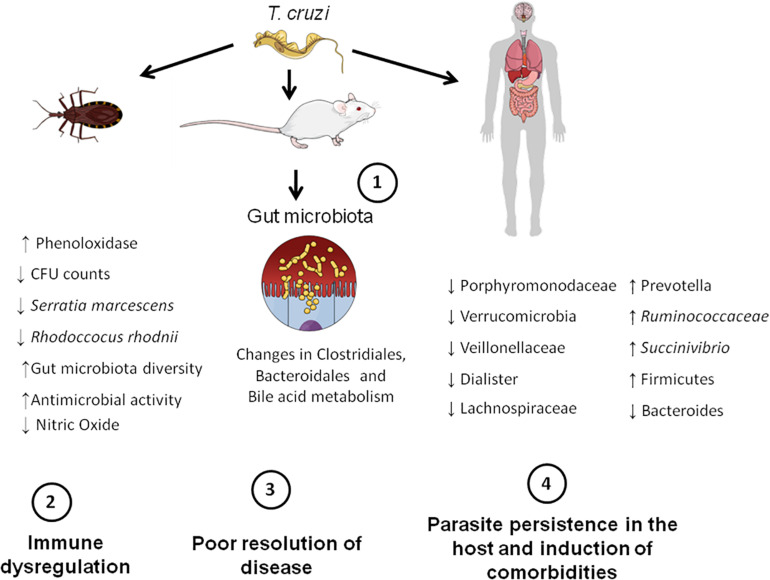
Microbiota-host communication pathways in CD. Firstly, *T. cruzi* establishes infection in the mammalian (mice and human) and insect hosts (insect vectors). This initial step triggers gut microbiota changes in the aforementioned hosts. For example, in insects, infection leads to a decreased number of bacterial species that possess trypanolytic activity, such as *Serratia marcescens*, which increases the susceptibility of the host to *T. cruzi* infection. In mice, changes in the order Bacteroidales and Clostridiales, as well in the bile acid metabolism take place. In humans, there is an overall increase in Firmicutes phylum and decrease in Bacteroides. These gut microbiota changes induced by the parasite lead to immune dysregulation and consequently poor resolution of disease. Finally, the parasite persists in the host and is able to induce comorbidities, such as major depressive disorder (MDD).

Here we provide pre-clinical and clinical evidence that gut microbiota may play a role in CD pathogenesis once it interferes with infection and its resolution. It became evident that *T. cruzi* induces changes in the gut microbiota and, especially in insects and mice, this is associated with a deregulated immune response and changed fecal metabolome, which might explain why the parasite persists in the host (Figure 2). However, the amount of data hitherto available is minute, and therefore, more studies are required to support the role of gut microbiota dysfunction in CD and its comorbidities. Future studies should test the effects of prebiotics and probiotics on preclinical and clinical CD to provide new treatment options for CD. Besides, they should investigate in depth the role of gut microbiota in CD pathogenesis, focusing on the role of microbes of certain microbial groups, such as Firmicutes and Bacteroidetes, and its impact in *T. cruzi* infection. Exploring the effects of *T. cruzi* infection in humans and what microbiota changes are caused by the parasite, as well as what species of microbes could be modulated to control disease are also necessary. Addressing the effects of different *T. cruzi* strains on microbiota changes as well as to explore the role of skin and oral microbiota in CD is also encouraged. Interestingly, other issues, such as the microbiota-gut-brain axis are currently underexplored in CD and thus deserve future attention.

A question that deserves attention is: is the dysfunction in the microbiota responsible for neuropsychiatric comorbidities observed in CD patients and could it be prevented or delayed by the use of gut microbiota-directed interventions? Answering this would allow us not only to better tackle CD but also its comorbidities. Moreover, given that pre- and probiotics are molecules derived from dietary compounds, such as vegetables and fruits, it is also essential to test whether a diet rich in these molecules could modulate CD and ameliorate disease state in the patients.

Other gut microbiota-targeted therapies that might have relevance to treating CD are fecal microbiota transplantation (FMT) and *synbiotics*. Although appealing, no data is hitherto available to support their use in CD patients. Furthermore, as there are considerable differences in the microbial profile of different portions of the gut (Donaldson et al., 2015), future studies should also employ colon biopsies to profile the microbial community in the intestinal mucosa of patients with CD.

As a matter of fact, funds for drug Research and Development (R&D) on CD are scarce, which happens for many reasons (Clayton, 2010), which are beyond the scope of this paper. Employing the suggestions mentioned above would probably draw the government and pharmaceutical companies’ attention to more funding for CD, probably leading to an increase in studies on the current topic and more elucidation on CD pathogenesis. As a consequence, CD would be less neglected than it is today.

## Author Contributions

ED-S conceived the study, performed the literature search, data collection, data analysis, wrote the manuscript, and created the figures under the supervision of CP. LM contributed to the writing of the manuscript. CP, GC, LM, and WS critically reviewed the manuscript. All authors approved the final version of this manuscript.

## Conflict of Interest

The authors declare that the research was conducted in the absence of any commercial or financial relationships that could be construed as a potential conflict of interest.

## Glossary

Germ-free (GF) mice – mice raised without a microbiome.

Y strain of *T. cruzi* – A strain that is associated with low parasitemia, high mortality in mice and that can also infect neurons and glia.

Trypomastigotes – The flagellated form of *T.cruzi*. Trypomastigotes are released in the insect vector feces when the insect takes a blood meal. They enter the host via mucosa or wounded sites and then infects mammalial cells, loses the flagellum and becomes amastigotes, which replicate by binary fission. Next, they turn back to trypomastigotes, causing cell lysis and spread of infection

Polysaccharide A (PSA) – a polysaccharide from the capsule of *B. fragilis* that has immunomodulatory effects.

Gnotobiotic mice – mice with only certain known bacterial strains/mice colonized with known bacterial strains.

Epimastigotes – non-infective form of *T. cruzi* detected in the gut of insect vectors. They replicate by binary fission. At a later stage during its life cycle, they become trypomastigotes.

Pathobiont – microorganisms of the microbiota with the potential to induce pathology (Round and Mazmanian, 2009).

Segmented filamentous bacteria (SFB) – gram-positive, spore-forming bacteria that modulates the immune system and attach to intestinal epithelium.

Antimicrobial peptides (AMPs) – peptides from the innate immune system with activity against viruses, fungi, parasites and bacteria.

Short-chain fatty acids (SCFAs) – bacterial metabolites, such as acetate, butyrate and propionate, derived from the fermentation of complex indigestible fibers.

Trimethylamine (TMA) – molecule that results from the metabolism of fat-rich or TMA-rich nutrients.

Trimethylamine-*N*-oxide (TMAO) – a microbial metabolite derived from metabolism of trimethylamine (TMA).

Enteric nervous system (ENS) – a network formed by neurons and glia in the gastrointestinal tract.

Prebiotics – non-digestible complex fibers that are fermented by the gut bacteria and promote the growth of some bacteria and health benefits.

Probiotics – consists of living microorganisms that confer health benefits when consumed in sufficient amounts.

Microbiota-gut-brain axis – the network composed of neural, immune and endocrine systems that mediate the effects of the gut microbiota in the host.

Fecal Microbiota Transplantation (FMT) – one type of microbiota directed intervention that consists in transferring faucal material from the donor to the recipient.

Secondary Bile Acids – molecules resulting from the microbes-driven metabolism of primary bile acids.

Synbiotics – association of prebiotics with probiotics.
